# Mechanisms underlying the development of type 1 diabetes in ART-treated people living with HIV: an enigmatic puzzle

**DOI:** 10.3389/fimmu.2024.1470308

**Published:** 2024-08-27

**Authors:** Silvere D. Zaongo, Abel W. Zongo, Yaokai Chen

**Affiliations:** ^1^ Department of Infectious Diseases, Chongqing Public Health Medical Center, Chongqing, China; ^2^ College of Food Science and Technology, Zhejiang University of Technology, Hangzhou, China

**Keywords:** HIV, antiretroviral therapy, ART, type 1 diabetes, T1DM, mechanisms

## Abstract

The immunopathogenesis of HIV infection remains poorly understood. Despite the widespread use of effective modern antiretroviral therapy (ART), people living with HIV (PLWH) are known to develop several comorbidities, including type 1 diabetes (T1DM). However, the etiology and critical mechanisms accounting for the onset of T1DM in the preceding context remain unknown. This article proposes to address this topic in order to provide further understanding and future research directions.

## 1 Introduction

The islets of Langerhans represent a critical segment of pancreatic biology, and as such, may be considered as the headquarters of pancreatic endocrine function. Indeed, they comprise various different cellular types, such as (i) alpha (α)-cells, which synthesize and secrete glucagon, (ii) beta (β)-cells, which produce insulin, (iii) delta cells, which secrete somatostatin, and (iv) P(F) cells, which produce pancreatic polypeptide (1). Thus, when β-cells either (i) do not function optimally and/or (ii) are damaged, diabetes mellitus may potentially develop. In the case of type 1 diabetes (T1DM), structural damage to β-cells occurs subsequent to immune responses which are directed against specific antigens on β-cells, or more specifically, against β-cell integrity (2). In other words, T1DM is an autoimmune disease which occurs subsequently to the destruction of pancreatic β-cells, and which is orchestrated by cell-mediated autoimmune responses (3). In the context of HIV infection, the mechanisms whereby the destruction of β-cells occur leading to the development of T1DM in ART-treated people living with HIV (PLWH) remains poorly understood.

This article reviews the potential immune mechanisms responsible for the onset of T1DM in HIV-infected individuals on ART. To this purpose, three parts to this article are presented. The first part establishes a relationship between HIV and T1DM through a review of the reported cases presented in the contemporary literature. The second part explores the immunobiology of T1DM, and represents a guide-map for the third part, which presents hypotheses which are formulated towards the mechanisms which may potentially be involved in the development of T1DM. The objective of this review is to provide a detailed look at the potential immunological mechanisms which underlie the onset of T1DM in PLWH on ART. This work is likely to be useful for the development of preventive approaches and future therapeutic solutions directed towards PLWH receiving ART.

## 2 Relationship between HIV and Type 1 diabetes

In the contemporary literature, case studies (**Table 1**) reveal that type 1 diabetes mellitus (T1DM) may develop in people living with HIV. On June 17th, 2024, 54 publications (case reports, meta-analysis, multicenter study, observational study, systematic review) were found on PubMed (https://pubmed.ncbi.nlm.nih.gov), using search terms such as “HIV”, “type 1 diabetes”, and “autoimmune diabetes”. The considered publications were written in English, and presented cases of HIV positive patients developing T1DM. From these results, nine publications (describing 11 cases) were selected, as they present cases of type 1 diabetes developing in ART-treated PLWH. Despite the very limited number of publications covering this research area, the contemporary literature informs us that immune reconstitution inflammatory syndrome (IRIS) (45.45%, 5/11) and Grave’s disease (36.36%, 4/11) are often also reported in ART-treated PLWH who subsequently developed T1DM, indicating that immunological mechanisms are likely to be involved in the development of T1DM in PLWH.

**Table 1:** Cases of type 1 diabetes diagnosed in ART-treated PLWH.

Author ^references^	Sex (age)	Antibodies reported	Diseases and/or comorbidities	Organs affected	CD4+ T-cells	ARTs (period)	BMI	IRIS
Taguchi et al. (4)	Male (38)	GADA, TSHR-Ab	Grave’s disease	Anal abscess	< 200 cells/μL to >300 cells/μL	RAL/TDF/FTC (48 months)	17.6	NR
Hughes et al. (5)	Male (48)	GADA	Graves’ disease, Hodgkin lymphoma	Right axillary mass	<100 cells/μL to 468-634 cells/μL	EFV/FTC/TDF (60 months)	NR	NR
Yeh et al. (6)	Male (36)	GADA, IA-2	Amoebic infection, syphilis, hepatitis B, and Grave’s disease	Gut, liver, thyroid gland	From 15.53 cells/μL to 429.09 cells/μL	BIC/FTC/TAF (9 months)	25.2	Yes
Takarabe et al. (7)	Male (30)	GADA, IA-2, TSHR-Ab, TPO-Ab, Tg-Ab	Hepatitis C, thyrotoxicosis	Liver, thyroid gland	From 12 cells/μL to 311 cells/μL	3TC/TDF/LPVr (18 months)	24.2	Yes
	Male (31)	GADA, TPO-Ab	Hepatitis C	Liver	From 14 cells/μL to 172 cells/μL	3TC/d4T/LPVr (10 months)	20.0	Yes
	Female (68)	GADA, IA-2, TPO-Ab, Tg-Ab	Grave’s disease	Thyroid gland	From 19 cells/μL to 316 cells/μL	3TC, ETR, RTV, DRV, RAL (55 months)	19.1	Yes
Mittal et al. (8)	Male (42)	NR	Severe autonomic neuropathy, gastroparesis	Pancreas transplant	From >250 cells/μL to 400 cells/μL	NVP/ABC/3TC (96 months)	25	NR
Genzini et al. (9)	Male (43)	NR	Terminal chronic renal insufficiency	Pancreas and Kidney transplant	803 cells/μL	NR		NR
Kamei et al. (10)	Male (40)	GADA	NR	NR	From 400-600 cells/μL to >1000 cells/μL	3TC/ABC/RAL (29 months)	<30	Yes
Shimoyama et al. (11)	Male (40)	GADA negative	Cytomegalovirus	Dysphagia (oropharyngeal cyst)	28 cells/μL	NR	18.8	NR
Bargman et al. (12)	Female (8)	GADA, IA-2, IR-Ab	Absence of serious infections or hospitalization.	NR	Normal CD4+ T-cell counts	3TC/ABC/d4T (72 months)	NR	No

3TC, Lamivudine; ABC, abacavir; BIC, Bictegravir; CNS, central nervous system; d4T, stavudine; DTG, dolutegravir; DRV, Darunavir; ETR, Etravirine; FTC, Emtricitabine; GADA, Glutamic acid decarboxylase autoantibody; IA-2, Islet antigen 2; IAA, Insulin autoantibody; IR-Ab, antiinsulin receptor autoantibody; LPVr, lopinavir boosted with ritonavir; NR, not reported; NVP, Nevirapine; RAL, Raltegravir; RTV, Ritonavir; TAF, Tenofovir alafenamide; Tg-Ab, antithyroglobulin antibody; TPO-Ab, antithyroid peroxidase antibody; TSHR-Ab, anti-TSH receptor antibody.

To elaborate further on the existing relationship between HIV infection and T1DM, findings and suggestions from previous publications are necessary. Thus, in their quest to elucidate possible mechanisms underlying the onset of T1DM during HIV infection, Takarabe et al., postulated two decades ago that ART (and the subsequent immune reconstitution associated with effective ART) may be implicated, without providing evidence of the possible underlying mechanisms involved (7). Further evidence indicates that the development of T1DM may be related to the presence of hyperinflammation (6). This hyperinflammatory state is referred to as IRIS, and occurs despite and as a consequence of the restoration of immune function (an increase in CD4+ T-cell counts and a suppression of HIV viral loads (13)). IRIS results from a fundamental imbalance between anti-inflammatory cytokines and proinflammatory cytokines which occurs rapidly after the recovery of immune function (14). Thus, ART induces a clinical improvement followed by a so-called ‘cytokine storm’, which manifests as increased levels of inflammation (with correspondingly higher levels of pro-inflammatory cytokines) (15). The incidence of IRIS is estimated at between 11.1% and 22.9% in HIV positive individuals receiving ART (16). Importantly, IRIS is known to induce health deterioration in PLWH, as the exaggerated inflammation resulting from its onset may target pathogenic microorganisms, non-pathogenic commensal organisms and latent organisms, and self-antigens.

**Figure 1:**
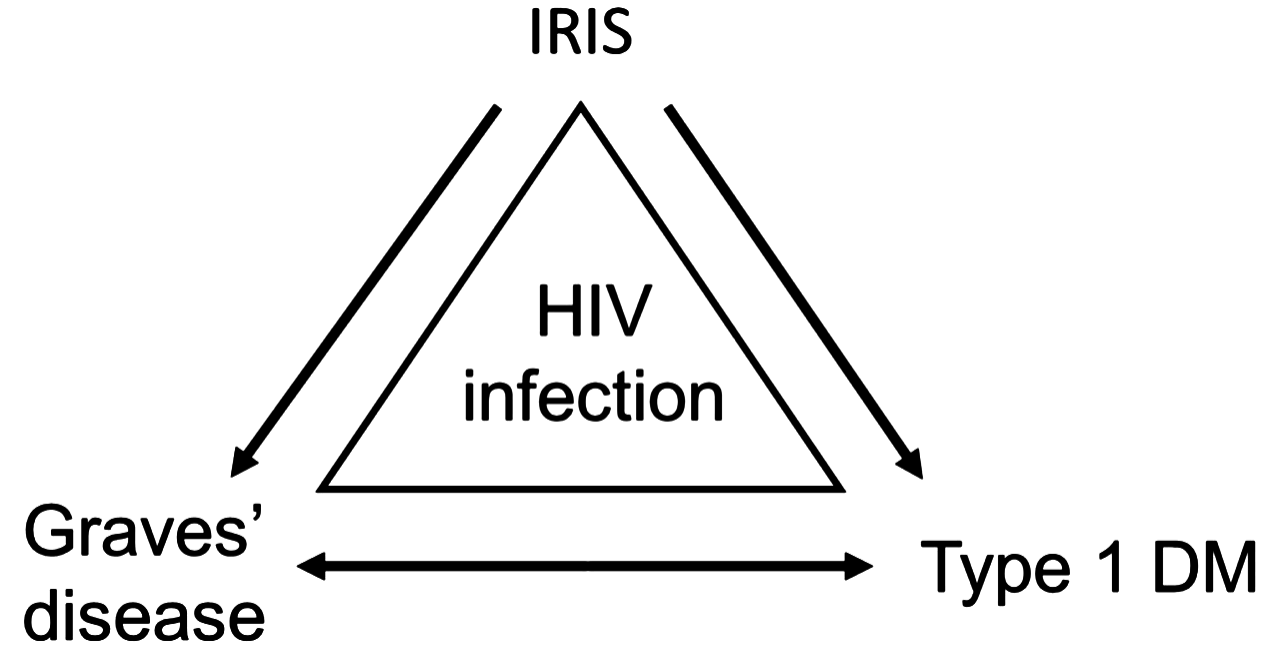
Relationship between HIV infection and T1DM. From the evidence recorded in **Table 1**, it is likely that other than Grave’s disease, opportunistic infections, autoimmune disorders, and hepatitis B or C infection may also be considered. From Yeh et al. (6). Reproduced under Creative Commons CC BY (CC BY 4.0).

In a recent publication, Yeh et al. (6), have proposed that the manifestation of IRIS may be responsible for T1DM development either directly or indirectly via a different disease, such as Grave’s disease (**Figure 1**). Among the cases reported in **Table 1**, it can be seen that the diagnosis of IRIS has been identified in six cases and remained unidentified in five cases. Interestingly, within the cases in which IRIS was diagnosed, 83.33% (5/6) of the cases developed T1DM. Even if the relationship between HIV and T1DM may be established through IRIS, the fundamental underlying immunological mechanisms leading to the emergence and manifestation of T1DM in ART-treated PLWH remain nebulous.

## 3 Immunological aspects of T1DM

Before further elaborating on the potential immunological mechanisms leading to the emergence and manifestation of T1DM in ART-treated PLWH, a clear understanding of the immunobiology of T1DM is necessary. Thus, a thorough review of the literature informs us that cells belonging to the innate and adaptative immune systems, in addition to the gut microbiome, are essential for this understanding.

### 3.1 Innate immunity

**Figure 2:**
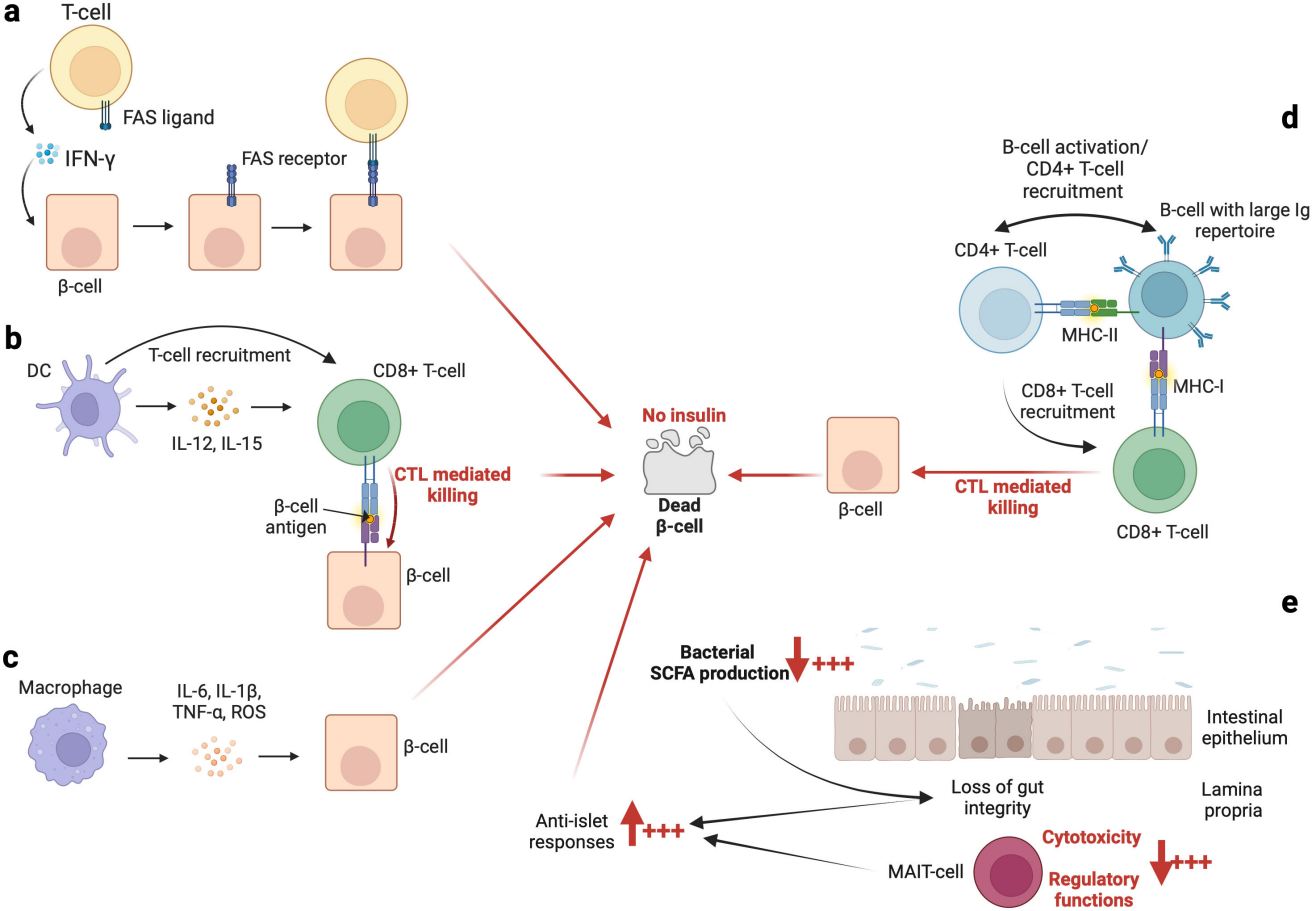
The immunological basis of autoimmune diabetes. An overview of the implications of some innate immune cells and the gut microbiome is summarized in **(A–E)**, respectively. In addition, the role of C-peptide should not be overlooked or underestimated. Indeed, as indicated by Washburn et al. (119), the presence of C-peptide contributes to (i) increase cells survival and (ii) decrease apoptosis process and reactive oxygen species (ROS) production. However, the levels of C-peptide are decreased in patients with T1DM which may favor β-cells death.

Contemporary literature informs us that antigen-presenting cells such as dendritic cells (DCs) in the pancreatic lymph nodes may secrete IL-12 and IL-15, which may activate autoreactive T-cells (17, 18). These T-cells may subsequently mediate the destruction of pancreatic β-cells either via major histocompatibility complex (MHC) class-I mediated cytotoxicity (CD8+ T-cells, **Figure 2B**) or via the secretion of interferon (IFN)-γ (by both CD4+ T-cells and CD8+ T-cells), which induces the expression of the death (apoptosis) receptor (i.e., FAS) on pancreatic β-cells (19). The CD4+ and/or CD8+ T-cell FAS-ligand may therefore activate apoptosis of β-cells through signaling of their FAS receptor (20) (**Figure 2A**). Furthermore, it has been demonstrated that a particular type of DC which produces IFN-γ, referred to as a plasmacytoid DC, may infiltrate the pancreas (under unclear circumstances), and may mediate diabetogenic T-cell responses (as indicated above), therefore favoring the development of T1DM (21). This has been observed in a murine model, and evidence suggesting the presence and role of this type of DC in humans remains to be demonstrated. Macrophages which reside in the pancreatic islets have also been suspected of causing β-cell depletion. Indeed, some researchers believe that macrophages may induce the production of IL-6 and reactive oxygen species through their ability to secrete tumor necrosis factor (TNF) and IL-1β (17, 22, 23) (**Figure 2C**). The role of macrophages in β-cell depletion has been further highlighted in investigations utilizing non-obese diabetic (NOD) mice, in which Calderon et al. (24), and Hu et al. (25), have demonstrated that depletion of macrophages that reside in pancreatic islets may prevent T1DM. Within the exocrine pancreas, there are neutrophils which may also contribute to the development of T1DM via the secretion of cytokines and chemotactic factors capable of modulating the activities of other immune cells (17, 26–28). Although they have been linked to T1DM pathogenesis (29, 30), the role of natural killer (NK) cells in T1DM development remains uncertain. Herold et al. (3), have postulated that a deeper understanding of the diversity of NK cell types may assist in further clarification of the precise role of each type of NK cell.

It appears that Toll-like receptors (TLRs), which are expressed on immune cells (including monocytes, DCs, macrophages, NK cells, T-cells, B-cells, and even on pancreatic β-cells) may also have the potential to serve as an indicator for T1DM. Researchers have reported that TLR4 expression on monocytes (31, 32) and CD4+ T-cells (33) in patients with T1DM is downregulated. Conversely, investigations by others (34, 35) have concluded that TLR4 and TLR2 levels on monocytes from T1DM patients are unregulated. To further illustrate the role of TLRs, it has been demonstrated that apoptosis of β-cells may be induced by the inflammatory response of macrophages through the TLR2/Myeloid differentiation primary response protein (MyD88)/Nuclear factor kappa-light-chain-enhancer of activated B-cells (NF-kB) signaling pathway (36).

Other than the cellular components of the innate immunity, it is worth mentioning the roles of pro-inflammatory cytokines such as IL-1 and the Type 1 interferons in the development of T1DM. Evidence indicates that compared to negative controls, patients with T1DM have higher serum levels of IL-1β (macrophage derived IL-1β) and IL-6 (37, 38). Furthermore, (i) higher expression of IFN response markers in islet cells and (ii) an IFN-induced gene signature preceding the emergence of islet autoantibodies have been reported in humans with T1DM (39). Additionally, IFN-γ and TNF for example, may induce expression of Class I and II human leukocyte antigen (HLA) molecules on β-cells (40), and the hyperexpression of HLA molecules enhances islet antigen recognition (41). Interestingly, it has been demonstrated that the onset of diabetes may be delayed by blocking the IFN-α receptor in NOD mice (42). This further emphasizes the role of IFN-α in the process of the establishment of T1DM. Also, IFN-α may promote both the presentation of self-antigens by islet cells and enhance islet cell detection by cytotoxic T-cells (39, 43). IFN-α participates in the recruitment of immune cells (including T-cells and NK cells) by inducing the secretion of various chemokines known to be involved in their recruitment (39, 43). Several researchers (44–46) have demonstrated that in patients with T1DM, there is higher expression of IFN-responsive genes well-known for their roles in apoptosis, endoplasmic reticulum stress, and antigen presentation. Notably, the tandem of IFN-α and IL-1β may act synergistically to provoke β-cell demise (44–46). Lastly, IFN-α may be involved in the T-cell-mediated elimination of pancreatic β-cells. Indeed, Chandra et al. (47), have observed that the knockout of the TYK2 gene [which originally encodes Janus kinase (JAK) and is known for being responsive to IFN-α] reduces human stem cell-derived islet sensitivity to T-cell-mediated destruction.

### 3.2 Autoantibodies and B cells

The role of B-cells and autoantibodies are intriguingly linked. Indeed, B-cells produce autoantibodies which assist in identification of the risk of autoimmune diabetes via the (i) capture and (ii) presentation of autoantigens to autoreactive T-cells (48, 49). In the period of time leading up to the drafting of this article, autoantibodies had not been considered to be pathogenic in the context of the development of T1DM (3). However, autoantibodies are useful in prediction of the progression of T1DM through the measurement of islet antibody levels and the specific antibodies which target islet cells (50, 51). In people presenting with a genetic predisposition to T1DM, the first autoantibodies targeting either glutamic acid decarboxylase (GAD) or insulin may be observed at 4–5 years of age. Nevertheless, they may appear earlier (at ages 1–2 years), and particularly those autoantibodies that are specific for insulin (52). Despite us being aware of the existence of these autoantibodies and their targets, the trigger that initiates the appearance of the first autoantibodies remains elusive. Some speculate that the trigger is possibly secondary to viral infection(s), and may be related to β-cell damage or stress. This theory is supported by the observations of Warncke et al. (53),, where it appeared that at around two months before seroconversion, a small increase in postprandial blood glucose may be detected. Thus, a possible association between β-cell perturbations and the initiation of autoantibody production may indeed exist.

While autoantibodies are not considered to be pathogenic, it has been demonstrated that B-cells play a critical role in the pathogenesis of T1DM, principally as antigen-presenting cells (**Figure 2D**). On the one hand, investigations (54–56) using animal models (NOD mice) have revealed that the loss of B-cell antigen presentation alone may effectively prevent T1DM. Conversely, the loss of antibody secretion has not been observed to be efficacious in prevention of the onset of T1DM (54–56). Furthermore, Silveira et al. (57), have demonstrated that B-cells having a highly restricted immunoglobulin (Ig) repertoire may induce significantly delays in the development of T1DM in NOD mice. Notably, Silveira et al. (57), have demonstrated that T1DM may be strongly suppressed when an Ig(mu)null mutation is introduced into B-cells. These modified B-cells have been subsequently unable to take up islet antigen via their receptor, and lack T-cell responses to the islet glutamic acid decarboxylase autoantigen (57). On the other hand, it has been observed that development of diabetes is enhanced when insulin is selectively recognized by B-cells (58). For example, Smith et al. (59), have observed that high-affinity insulin-binding B-cells occur exclusively in the anergic naïve compartment (characterized as IgD+IgM- B-cell). However, they have observed that patients with T1DM (prior to and at the time of diagnosis) display a loss of anergy of high-affinity insulin-binding B-cells (59). Other than antigen presentation and the selective recognition of insulin, age-related infiltration of B-cells into the islets and mediated elimination of β-cells may be additional mechanisms which explain the onset of T1DM. Indeed, Leete et al. (60), have observed that compared to older T1DM patients, the islets of those below 7 years of age contain (i) a significantly higher number of infiltrated B-cells and (ii) fewer remaining β-cells.

### 3.3 Implications of T-cells

Recently, Herold et al. (3), have posited that the role of T-cells in the development of T1DM had been suspected four decades ago. Today, autoreactive T-cells may be considered as the main effector cell of β-cell autoimmunity (61). As such, past studies have observed that the suppression of T-cell receptor (TCR) signaling when using cyclosporin A [an immunosuppressive drug, use of which has now been restricted due largely to toxicity concerns (62, 63)] helped to (i) promote the preservation of β-cell function and (ii) reduce exogenous insulin supplementation. Similarly, one study enabling T1DM pancreatic tissue profiling has revealed that both CD4+ and CD8+ T-cells are recruited simultaneously in the islet (64). Thus, Damond et al. (64), have noted that T-cells induce insulitis, particularly in islets that retain insulin-producing β-cells. Notably, Willcox et al. (65), and subsequently Campbell-Thompson (66) have observed that human insulitis generates more CD8+ T-cells than CD4+ T-cells. Importantly, and for a thus far unexplained reason, not all islets within the pancreas are affected equally, as some islets may be free of immune cells while others display large numbers of infiltrating T-cells (3).

It has been demonstrated that CD4+ T-cells contribute to pancreatic β-cell death via (i) the production of cytokines (IFN-γ and TNF-α), (ii) the stimulation and formation of M1-like macrophages, (iii) the promotion of DCs which in turn enhance CD8+ T-cell responses, and (iv) the activation of B-cells (67–70). The peculiarity of CD4+ T-cells isolated from the pancreas of T1DM patients is that they are able to specifically recognize and target proinsulin, GAD, and insulinoma-associated protein 2(IA2) (71–73). In the peripheral blood, it has been observed that levels of activated (CD38+) islet antigen-specific memory CD4+ T-cells are elevated in T1DM patients (74). These cells notably display reactivity against GAD65, preproinsulin (PPI)_78-90_, islet-specific glucose-6-phosphate catalytic subunit-related protein (IGRP), PPI_35-47_, chromogranin A (ChgA), and zinc transporter 8 autoantibody (ZnT8) (74). Moreover, CD4+ T-cells may be stimulated by the insulin and/or insulin peptide fragments released by β-cells into the bloodstream, regardless of the relative distance between the β-cell and CD4+ T-cell location (75). With respect to the phenotype of CD4+ T-cells encountered most frequently in T1DM patients, it was initially believed that the T helper 1 cell (T_h_1) was more prevalent, as T_h_2 cells were thought to have a more protective function (76). However, new evidence gleaned from the contemporary literature suggests that it is not as simple as it once seemed (76). Indeed, T-cell differentiation is likely to be even more diverse than was previously appreciated, and as such, a pathogenic role of the T_h_17 cell has also now been documented (77, 78). Researchers have also observed that a subset of CD4+ T-cells, referred to as T_h_40 cells (CD3+CD4+CD40+) and found in the peripheral blood of humans, are significantly increased in number in T1DM patients relative to numbers in healthy controls, suggesting their potential utilization as biomarkers (79). Similarly, T-cells having the follicular helper (T_fh_) phenotype expressing elevated levels of IL-21 have been reported in patients with T1DM (80–82). Moreover, Ekman et al. (83), have observed that levels of T peripheral helper (T_ph_) cells (which act in peripheral tissues and have a similar phenotype as T_fh_ cells) are increased in high-risk individuals who progress to T1DM and also at TD1M onset. Interestingly, T_fh_ and T_ph_ cells may interact with B-cells to form aggregates referred to as tertiary lymphoid organ (TLO)-like structures (84). These TLO-like structures have been identified in the pancreas of NOD mice (85). In humans, their presence in the pancreas has not definitively been recognized; however, one recent study has demonstrated that out of 21 organ donors with T1DM, 12 individuals had evidence of pancreatic TLOs (86).

Evidence shows the presence of autoantigen-reactive CD8+ T-cells in the pancreas [particularly an enrichment of islet autoantigens, such as ZnT8_186-194_ (87)] and in the peripheral blood of people with T1DM (88, 89). Interestingly, it is also recognized that (i) 60-70% of patients with T1DM have islet infiltrating preproinsulin (PPI)-reactive CD8+ T-cells and (ii) *in vitro* analysis has revealed that PPI-reactive CD8+ T-cells may destroy human β-cells (90). Other than the presence of PPI-reactive CD8+ T-cells in patients with long standing T1DM, various other types of specific autoantigens may be found within the same islet (91). Moreover, the frequency of some types of reactive CD8+ T-cells is associated with insulin-derived substrate. As such, levels of C-peptide (a substance that is created when hormonal insulin is produced and released into the body) have been positively associated with the frequency of CD57+ effector memory islet-specific CD8+ T-cells (92). The level of C-peptide is critical, as researchers have observed that between 20 to 50% of patients (depending upon the study) maintain long-term C-peptide levels (93–95). In these individuals it has been observed that with sustained C-peptide levels, T1DM had a later age of onset, while individuals who had a relatively younger onset of T1DM did not show sustained C-peptide levels (93, 94). Furthermore, Wiedeman et al. (96), have demonstrated that autoreactive CD8+ T-cell exhaustion, with expression of eomesodermin (EOMES), programmed cell death (PD)-1, T-cell immunoreceptor with Ig and ITIM domains (TIGIT), CD244 (also known as 2B4), and CD160 may be used to discriminate subjects with slow T1DM progression. It is also worth noting that researchers have also observed islet-reactive CD8+ T-cells in healthy individuals (87, 96). Thus, the question as to what may trigger the pathological functions of these cells remains to be answered. It has been suggested that antigen peptides from the gut microbiome might modulate CD8+ T-cell activity and trigger either beneficial or pathological functioning of these cells (97).

In addition to CD4+ and CD8+ T-cells, the role of T-regulatory (Treg) cells [whose primary function is to suppress the immune response and to maintain homeostasis and self-tolerance (98)] in the development of T1DM is worthy of mention. It has been suggested that in T1DM, (i) Treg cells may be dysfunctional and/or (ii) memory effector T-cells exhibiting diabetogenic responses may resist elimination of Treg cells (99). Potentially compromised signaling by IL-2R (the mechanism whereby the metabolic and functional activities of Treg cells are ensured) in patients with T1DM may explain the lack of efficacy of Treg cells (100). Indeed, on the one hand, Garg et al. (101), have demonstrated that an insufficient forkhead box protein 3 (FOXP3 or scurfin) expression in Treg cells may be a potential consequence of defects in the IL-2R pathway. On the other hand, the preceding authors have also observed that insufficient FOXP3 expression may be the consequence of a reduced phosphorylation of STAT5 (101). That said, it is also known that Treg cells display aberrant behavior (instability) during T1DM, as they produce IFN-γ (102). This instability of Treg cells may be maintained by IL-12, IL-23, and IL-21 (102, 103) secreted by T_FH_ cells, indicating their expansion in secondary lymphoid organs (104).

### 3.4 Gut microbiome and T1DM

It is likely that the emergence of T1DM may be linked to alterations in the core composition of the gut microbiome. Indeed, fecal transplantation from healthy donors to patients with recent-onset (<6 weeks) T1DM has been observed to preserve residual β-cell function (105). As fecal transplantation is able to abolish the decline in endogenous insulin production, de Groot et al. (105), have suggested that the gut microbiome and its associated metabolites may play significant roles in the pathogenesis of T1DM. The enigmatic puzzle related to the role of the gut microbiome in T1DM remains to be investigated; however, small fragments of intriguing information related to this relationship are able to be gleaned from the contemporary literature; for example, observations from the analysis of stool samples have shown that T1DM may linked to certain enterovirus infections (106). These findings indicate an association between persistent enterovirus infection and islet autoimmunity, and progression of T1DM (106). Interestingly, Paun et al. (107), have observed that dysfunction of gut-associated lymphoid tissues (including invariant immune cells such as T-cells, innate lymphoid cells, and others) and loss of microbiome diversity may provoke a loss of tolerance towards the commensal gut microbiome, and thus induce exaggerated levels of inflammation. Consequently, the activation of the immune system through autoreactive cells and/or immune cells in general may induce cross-reactivity between autoantigens and the commensal gut microbiome (107). To further illustrate the point regarding the activation of the immune system against the commensal gut microbiome, it is worth noting that in individuals with T1DM or in those at risk of T1DM, antibodies against commensal microorganisms have been identified (108). Furthermore, it is known that short-chain fatty acids (SCFA) produced by commensal bacteria may influence systemic immune regulation (109–111). For example, in instances of dysbiosis leading to low levels of SCFA, the cytotoxicity and regulatory functions of mucosal-associated invariant T (MAIT)-cells are altered, which facilitates (i) the loss of gut integrity, (ii) an increased anti-islet response, and (iii) the development of T1DM (112) (**Figure 2E**). As such, Rouxel et al. (112), have suggested that MAIT-cells may represent a novel biomarker for T1DM, and that MAIT-cell monitoring may become a necessity for at-risk individuals in the future.

Notably, observations from Demirci et al. (31), indicate that the stool of patients with T1DM contain high amounts of *Bacteroidetes* and *Firmicutes*. Comparatively, healthy individuals have significantly lower amounts of these commensals. In parallel, T1DM patients display downregulated and upregulated levels of TLR2 and TLR4, respectively. The preceding authors have suggested that high levels of *Bacteroidetes* and *Firmicutes* in the gut microbiome may modulate the pathogenesis of T1DM through TLR4 and TLR2 (31). In addition to the preceding evidence, Lee et al. (36), have observed (i) that the macrophage response to inflammation (via the TLR2/MyD88/NF-kB pathway) may induce apoptosis in pancreatic β-cells and (ii) that via TLR2-dependent antigen-presenting cell activation, late apoptotic pancreatic β-cell destruction may sensitize the development of T-cells which mediate diabetogenic responses. Thus, the importance of TLRs in the relationship between the gut microbiome and T1DM warrants further scrutiny.

Cumulative evidence also indicates that patients with T1DM have increased intestinal permeability (113, 114), also referred to as a ‘leaky gut’. Thus, the pathogenesis of T1DM may potentially be induced via translocated microbes and their associated metabolites. As demonstrated by Costa et al. (115), and Alkanani et al. (116), gut microbes and their associated metabolites may be transported from the bloodstream into pancreatic lymph nodes, where they may trigger NOD2 activation, the TLR2/MyD88/NF-kB pathway, or the TLR3/MyD88 pathway, all of which may contribute to the destruction of pancreatic β-cells. In the duodenum of patients with T1DM, the activation of innate and adaptive immunity has been observed. This profile may disrupt intestinal epithelial/lymphoid cellular function (by production of mucin and β-defensins) and promote the expansion of autoreactive T-cells (117). Also, it is important to recall for example, that β-defensin 14 from intestinal lymphoid cells (and stimulated by the gut microbiome) has been observed in pancreatic endocrine cells (118). Interestingly, it seems that β-defensin 14 may stimulate TLR2 on macrophages, which in turn recruits protective Treg cells to maintain immune tolerance within the pancreas (118). From this picture, one may speculate that in the context of the leaky gut (associated with disrupted intestinal epithelial/lymphoid cellular function) seen in patients with T1DM, protective defenses which maintain the integrity of the pancreas are likely to be subverted. Future studies in this realm of investigation are warranted.

A summary of the immunobiology of T1DM is provided in the figure below (**Figure 2**).

## 4 Hypotheses of potential underlying immune mechanisms

### 4.1 T-cells and their crosstalk with other immune cells

**Figure 3:**
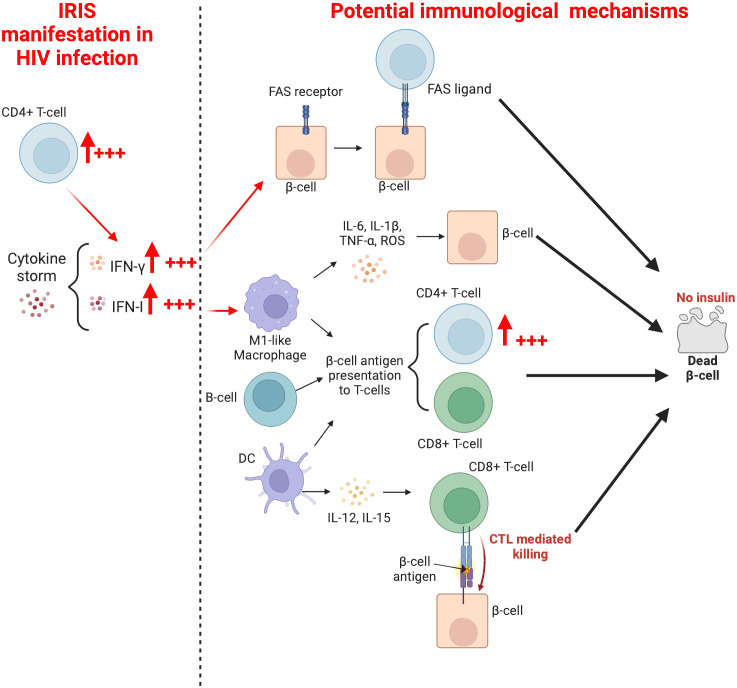
Mechanisms whereby a rapid increase in CD4+T-cells may mediate the development of T1DM in ART-treated PLWH.

The causes of T1DM in ART-treated PLWH are unknown. However, in their study, Yeh et al. (6), have noted that free thyroxine (FT4) and hemoglobin A1c (HbA1C) levels correlate positively with CD4+ T-cell counts. This is a potential hint that suggests the role of CD4+ T-cells in the development of T1DM. It is likely that IRIS, which is the key factor in the relationship between HIV and T1DM, favors the immunologically overenthusiastic reactive activity of T-cells in general, and CD4+ T-cells particularly (**Figure 3**). Interestingly, during HIV infection, the production of IFN-γ by T-cells is well documented (120–123), and subsequent to ART initiation, the dramatic change in CD4+ T-cell count (from extremely low to rapid recovery, **Table 1**) may provoke a cytokine storm (including significantly elevated levels of IFN-γ). Thus, as indicated in the point related to the immunology of T1DM, IFN-γ may induce the expression of the FAS death receptor on pancreatic β-cells (19). This phenotype exhibited by β-cells may be regarded as a ticking bomb, as upon attachment to the CD4+ T-cell FAS-ligand, the apoptosis and death of the β-cell will inevitably follow (20). In addition, IFN-γ may induce the expression of P-selectin glycoprotein ligand (PSGL-1) on immune cells (including T-cells) (124). In turn, PSGL-1 could possibly favor their infiltration into islets of Langerhans (via a process referred to as extravasation) subsequent to PSGL-1 engagement with P- or E-selectin on platelets and endothelial cells, respectively) where they may mediate diabetogenic responses against β-cells. However, the latter assertion requires robust research evidence to become universally acceptable. Altogether, it is likely that a therapeutic option which targets IFN-γ, as suggested by De Benedetti et al. (125), may potentially help in the protection and preservation of β-cells in PLWH receiving ART. Lastly, the utilization of adjunctive therapeutic options such as metformin, which significantly reduces inflammation (126), may encourage protective conditions to mitigate the onset of T1DM in ART-treated PLWH.

In the context of HIV infection, M1-like macrophages may be stimulated after HIV recognition by TLR7 (127, 128) or by direct contact and subsequent fusion of an infected CD4+ T-cell with a macrophage (129). This promotes antigen presentation, T-cell activation, and massive IFN-I secretion to induce innate and adaptative immune responses during the early phase of infection (130). However, due to the depletion of immune cells (T-cells in particular, and also other target cells) subsequent to HIV infection and the progressive diminution of proinflammatory cytokines promoting the expansion of M1-like macrophages, there is an establishment of an IL-4- and IL-13-rich environment responsible for the switch from the M1 to the M2-like macrophage, which possesses anti-inflammatory properties (reduced or undetectable levels of IFN-I) (131). One may speculate that the rapid increase in CD4+ T-cell numbers associated with initiation of ART, in conjunction with a latent or diagnosed disease/infection in the context of a hyperinflammatory state may induce another switch from the M2-like macrophage to the M1-like macrophage. The latter may mediate pancreatic β-cell antigen presentation to activated CD4+ and CD8+ T-cells, leading to pancreatic β-cell destruction and progression of T1DM. As it has been demonstrated that the depletion of macrophages residing in the pancreatic islets of mice may prevent T1DM (24, 25), macrophage (particularly M1-like macrophage) depletion in the pancreatic islets of PLWH may well prevent β-cell destruction. The utilization of anti-IFN-γ and anti-TNF-α may also prevent the expansion of the M1-like macrophage, and ultimately contribute to the preservation of β-cell integrity.

DCs, which are among the first cells to encounter HIV in the body (132), are activated after HIV recognition by TLR7 or through CD4, CXCR4, and CCR5 receptors (133). During HIV infection, the number of DCs in peripheral blood decreases as the number of CD4+ T-cells diminish, either because of the increased rate of cellular death or due to the migration of DCs to lymph nodes, where they accumulate, remain activated, and eventually undergo apoptosis (134, 135). *In vivo* investigations have reported that DCs sampled from elite controllers are relatively more competent at mediating T-cell responses compared to DCs from other categories of HIV-infected individuals (136). In the context of this article, it is possible that hyperinflammation and the rapid CD4+ T-cell gain after ART initiation dysregulates DC activities in the pancreatic lymph nodes (manifested by enhanced secretion of IL-12 and IL-15). Consequently, DCs morph into highly efficient antigen presenting cells, which recruit further T-cells to mediate diabetogenic responses. Only future studies will unearth new information to either support or refute this hypothesis. Besides, two subsets of DCs (myeloid DCs and plasmacytoid DCs) have been described (137), and the potential role of each of these in the onset of T1DM subsequent to ART administration in PLWH requires elucidation. In the same line and related to antigen presenting cells, the rapid increase of CD4+ T-cell numbers may also trigger the pathogenesis of T1DM in PLWH via activation of B-cell activities (antigen presentation, immunoglobulin production, and/or insulin-binding affinity) (**Figure 3**). Future studies are required to unravel the role played by the crosstalk between CD4+ T-cells and B-cells in the preceding context.

Furthermore, some researchers (138–141) have postulated that ART, in addition to favoring an increase in CD4+ T-cell numbers, favors a rapid increase in memory CD4+ T-cell counts. As a consequence of effective ART, HIV viral load decreases and apoptosis of immune cells decreases in parallel. Thus, the numbers of existing memory CD4+ T-cells may increase via the phenomenon of clonal proliferation. Comparatively, there is a slower increase in numbers of naïve CD4+ T-cells. The preceding scenario may also occur with CD8+ T-cell numbers. Over time, ART therefore actively participates in the increase in CD4+ and CD8+ T-cell numbers (particularly memory T-cells) as well as B-cell numbers (142), which is likely to account for the substantially improved cell-mediated and antibody-mediated immunity seen in HIV-infected patients responding to ART (14). Unfortunately, the rapid increase in CD4+ and CD8+ T-cell counts may lead to (i) an excessive and inappropriately focused pathogen-specific cellular immune response, (ii) a decrease in the capacity of regulatory T-cells to effectively regulate and attenuate the resultant inflammation, (iii) an uncoupling of innate and acquired immunity. All of the preceding mechanisms may be exacerbated in the context of IRIS and further promote the pathogenesis of T1DM in ART-treated PLWH.

### 4.2 The gut microbiome

**Figure 4:**
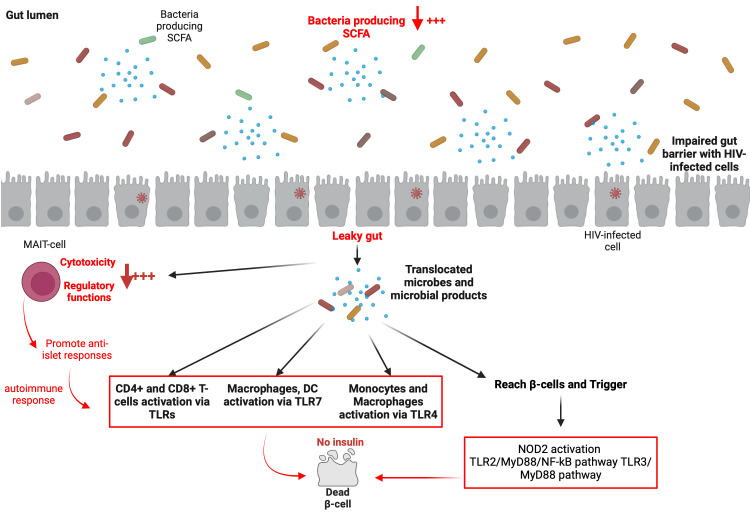
Potential major roles played by the gut microbiome in the development of T1DM in ART-treated PLWH.

The complete disruption of normal gut homeostasis during HIV infection has been well documented by our group (143–145) and others (146–148). While HIV-negative individuals possess a regulated microbiome and preserved gut integrity, HIV-infected individuals present both an HIV-associated gut dysbiosis syndrome (a perturbation of gut microbiome composition favoring the establishment and disproportional growth of pathogenic microbes) and a leaky gut syndrome (149, 150). Thus, in HIV-infected individuals, there is a persistent inflammation resulting from the leaky gut whereby gut microbes and their metabolites/toxins (LPS, β-glucan) are translocated into the circulating blood. One may legitimately point out the potential role of the preceding factor in the development of T1DM, particularly if it occurs in circumstances of hyperinflammation subsequent to a rapid CD4+ T-cell increase (**Figure 4**). Indeed, the leaky gut may induce monocyte and macrophage activation via TLR4, leading to overexpression of IL-6, sCD14, and sCD163 (151). Notably, upregulation of TLR4 has been reported in patients with T1DM (31). This suggest that gut microbes and their metabolites/toxins (microbes/metabolites/toxins) may mediate T1DM via two mechanisms. Firstly, by direct contact of gut microbes/metabolites/toxins with pancreatic β-cells within the islet of Langerhans, where the microbes/metabolites/toxins may trigger NOD2 activation, the TLR2/MyD88/NF-kB pathway, or the TLR3/MyD88 pathway in order to destroy pancreatic β-cells, and secondly, by promotion of the activation of immune cells (such as CD4+ T-cells, CD8+ T-cells, macrophages, or DCs) via TLRs, which subsequently mediate pancreatic β-cell demise. In the specific context of HIV infection, the precise TLRs involved, the cell types implicated, and the microbes/metabolites/toxins associated with T1DM progression need to be accurately determined in future studies. The underlying processes whereby immune cells fail to discriminate self- from non-self-antigens in this context remains to be determined as well. Nevertheless, it is known that low production of SCFA within the gut provokes an alteration of MAIT-cell functions (cytotoxicity and regulatory functions), and may increase anti-islet responses (112). Low levels of SCFA are also reported in HIV-infected individuals (152–154) due to the diminution of beneficial bacteria such as *Akkermansia muciniphila*, *Bacteroides*, *Bacteroides vulvae*, *Diplococcus*, and *Arbuscular roseus* (144). Therefore, the monitoring of MAIT-cells and their functions may reveal significant knowledge that may well be important in the understanding of the development of T1DM during HIV infection.

### 4.3 Vitamin D deficiency

In addition to the preceding potential factors, the role of vitamin D should not be overlooked. As indicated by Wang et al. (155), PLWH are at higher risk of having vitamin D deficiency compared to the general population [odds ratio of 1.502 (95% CI, 1.023–2.205; *p*=0.038)]. Interestingly, the risk is even higher in ART-experienced PLWH [odds ratio of 2.296 (95% CI, 1.287–4.097; *p*=0.005)] (155). This observation is significant, as cumulative evidence suggests that vitamin D deficiency is positively associated with T1DM (156–159). It has been shown that vitamin D may (i) regulate β-cell activity, (ii) increase insulin sensitivity, and (iii) protect islet cells by reducing the expression of inflammatory factor-induced apoptosis gene-related proteins (160). In a context of vitamin D deficiency, impairment of the transcription of islet cell function genes and abnormal glucose tolerance may be observed (161), and may potentially lead to an increased risk of developing T1DM (157).

It is known that vitamin D has immunoregulatory functions and anti-inflammatory effects (157, 162), which are mediated through vitamin D receptors present not only on islet β-cells, but also on immune cells. As such vitamin D may (i) regulate T-cell activity, (ii) promote CD4+ T-cell differentiation into Th2 and Treg cells, (iii) curb the production of Th1 and Th17 cells, (iv) stimulate the release of anti-inflammatory cytokines, and (v) reduce the production of proinflammatory cytokines (including IFN-γ and TNF-α). Furthermore, vitamin D, while promoting monocyte maturation into macrophages, decreases the monocyte ability to present antigens to T-cells. Indeed, vitamin D may reduce the superficial expression of MHC-II (163). Vitamin D also impairs the maturation of DCs and mediates the production of DCs which are unable to present antigens, as they do not possess surface MHC molecules (164).

It is thus likely that the deficiency of vitamin D observed in PLWH may promote T1DM secondary to the attenuation of immunoregulatory and anti-inflammatory functions of vitamin D.

## 5 Conclusion and perspectives

The immunobiology of type 1 diabetes in HIV-infected individuals on ART remains poorly described, documented, and understood. Interestingly, it seems that IRIS associated with the presence of coinfections (hepatitis B or C, opportunistic infections) and/or comorbidities (autoimmune diseases, non-communicable chronic diseases) may trigger immune responses favoring the progression to T1DM. With an overview of the immune mechanisms that underpin the pathogenesis of T1DM, this article also presents hypotheses for the potential mechanisms that underpin the complex autoimmune manifestations in HIV-infected individuals on ART. T-cells (particularly CD4+ T-cells) are likely to be the major cellular element to consider in the quest to understand the pathogenesis of T1DM in PLWH. The positive correlation of CD4+ T-cell counts with FT4 and HbA1C levels, and the ability of CD4+ T-cells to modulate the activities of other innate immune cells, especially in the hyperinflammatory context of IRIS, should not be underestimated. The presence of autoimmune disease such as Grave’s disease may favor the loss of tolerance to some self-antigens, leading to the development of T1DM (165); however, to understand the capacity of T-cells and other immune cells to discriminate self- and non-self-antigens, it is essential to understand the integrity of the thymus in PLWH who develop T1DM after ART initiation. Indeed, at the primary level, the tolerance of T-cells to self-antigens is modulated by the thymus (166). Therefore, future studies investigating the role of the thymus in the development of T1DM in PLWH after ART initiation are also warranted. Lastly, this article reports that the gut microbiome may be the second element to consider in the development of T1DM in PLWH. The gut microbiome has the capacity to modulate the activities of immune cells via TLRs, and to attenuate their ability to discriminate self- and non-self-antigens. In this regard, in-depth investigations with respect to the role of MAIT-cells are necessary in the context of HIV infection. Other varieties of cells that potentially play similar roles require identification and classification in further future studies.

Despite the extensive information collected and collated from the contemporary literature, it is worth mentioning that most of the studies covering the immunological aspects of T1DM have been conducted in murine models, and very few relevant studies have been conducted in human cohorts. This represents a significant limitation, and should be considered in future investigations in PLWH.
